# Space Environment Impacts Homeostasis: Exposure to Spaceflight Alters Mammary Gland Transportome Genes

**DOI:** 10.3390/biom13050872

**Published:** 2023-05-22

**Authors:** Osman V. Patel, Charlyn Partridge, Karen Plaut

**Affiliations:** 1 Cell and Molecular Biology Department, Grand Valley State University, Allendale, MI 49401, USA; 2 Annis Water Resources Institute, Grand Valley State University, Muskegon, MI 49441, USA; 3 Department of Animal Sciences, Purdue University, West Lafayette, IN 47906, USA

**Keywords:** mammary gland, metabolite, microgravity, pregnancy, rat, transportome

## Abstract

Membrane transporters and ion channels that play an indispensable role in metabolite trafficking have evolved to operate in Earth’s gravity. Dysregulation of the transportome expression profile at normogravity not only affects homeostasis along with drug uptake and distribution but also plays a key role in the pathogenesis of diverse localized to systemic diseases including cancer. The profound physiological and biochemical perturbations experienced by astronauts during space expeditions are well-documented. However, there is a paucity of information on the effect of the space environment on the transportome profile at an organ level. Thus, the goal of this study was to analyze the effect of spaceflight on ion channels and membrane substrate transporter genes in the periparturient rat mammary gland. Comparative gene expression analysis revealed an upregulation (*p* < 0.01) of amino acid, Ca^2+^, K^+^, Na^+^, Zn^2+^, Cl^−^, PO_4_^3−^, glucose, citrate, pyruvate, succinate, cholesterol, and water transporter genes in rats exposed to spaceflight. Genes associated with the trafficking of proton-coupled amino acids, Mg^2+^, Fe^2+^, voltage-gated K^+^-Na^+^, cation-coupled chloride, as well as Na^+^/Ca^2+^ and ATP-Mg/P*i* exchangers were suppressed (*p* < 0.01) in these spaceflight-exposed rats. These findings suggest that an altered transportome profile contributes to the metabolic modulations observed in the rats exposed to the space environment.

## 1. Introduction

The new era of public-private partnership to explore space marked a significant milestone in 2020 with the first successful launch of astronauts into the low-earth orbit aboard a commercially developed spacecraft [1,2]. This accomplishment came almost a decade after the National Aeronautics and Space Administration (NASA) officially retired the Space Shuttle program after 30 years of missions [1,2]. With this collective achievement, NASA plans to send, within the next decade, manned missions that are almost 1000 (Moon) to 100,000 (Mars) times farther away than the low earth orbiting International Space Station (ISS) [3,4]. Additionally, to advance the understanding of the solar system, NASA intends to establish a permanent lunar outpost to serve as a segway to deep space exploration [3,4]. However, the continuous inhabitation of ISS by humans for over two decades has revealed that *g*-load variation significantly impacts homeostasis [3,5,6,7,8]. Therefore, to establish and maintain sustained human presence beyond the earth’s lower orbit, it is critical to comprehensively identify the physiological perturbations induced by the space environment and develop effective countermeasures.

Earth’s gravity has played a central role in the structural and functional evolution of the diverse human organ systems and overall homeostasis [9,10]. For instance, the cardiovascular architecture and regulatory mechanisms have evolved to respond directly to the earth’s force of gravity and sustain homeostasis via hemodynamic modulations [11,12]. Similarly, the musculoskeletal system has evolved to counter the gravitational force through structural, as well as functional adaptations and contribute to homeostasis through thermoregulation and governing locomotion [13,14]. Conversely, the low gravity environment of space affects the microstructural characteristics of the circulatory system along with peripheral vascular resistance [15,16]. Similarly, the lowered force of gravity leads to pronounced bone loss stemming from the disruption of the intricate equilibrium of bone cells that regulate bone growth, shape, and structural integrity [17,18]. The space environment also impacts the morphology and structural properties of the skeletal muscles leading to atrophy, diminished force, and loss of functionality [17,19]. Overall, exposure to the space environment has been shown to affect multiple organ systems that plausibly perturbs homeostatic balance in humans [3,10,20]. However, more studies are needed to evaluate the long-term adverse effects of space exposure on diverse organ systems.

The mammary gland is a unique organ such that much of its development is postnatally, and it undergoes dynamic transformations in terms of mass, structure, and composition during the lifetime of a female [21,22,23]. For example, isometric growth and pronounced ductal arborization take place during the peripubertal window [24,25]. While dramatic allometric expansion, pronounced morphogenetic transformation and differentiation of the secretory alveolar lineage actuated by intrinsic and systemic mammogenic factors happen primarily during pregnancy [24,25]. Animal models, particularly murine, have been the predominant mammalian models for studying homeostatic adaptations induced by the space environment, with the first mouse launched into space as far back as the 1950s [9]. Accordingly, it is important that the physiological aberrations observed with murine studies are thoroughly investigated and risk mitigation strategies developed to maintain optimal astronaut health during deep-space missions.

In recent decades, the critical physiological role of ion channels and membrane proteins that facilitate a diverse array of molecules across cell and organelle membranes is well recognized [26,27,28]. These specialized proteins involved in the intricate translocation of molecules are broadly subdivided into four families; ATPases, ATP-binding cassettes (ABC), solute carrier proteins (SLC), and ion channels [26,27,28]. Collectively, these membrane-spanning proteins play a vital role in maintaining homeostasis by not only regulating the intra- and extra-cellular exchange of ions, metabolites, and nutrients but also affecting water uptake, pH, and global cellular volume [29,30]. Dysregulation of these proteins is widely implicated in diverse pathological conditions from metabolic to neurodegenerative, as well as cancer [31,32,33,34,35]. Comparably, the space environment causes dynamic adjustments across major anatomical systems from cardiovascular to digestive to reproductive [7,17,18,36,37,38,39,40,41,42,43,44,45,46,47,48]. However, none of the studies have described in detail the role of transmembrane transporters in the observed physiological adaptations. Previously, we used a comparative gene expression approach to characterize the complex homeorhetic adaptation across key metabolic tissues during pregnancy-to-lactation evolution [49], and the expression divergence of the SLC transporters [50] employing a rat mammary model. Additionally, we also showed how gravity affected circadian synchronization, metabolic and energy homeostasis [51]. However, to our knowledge, there is no report on the effect of the space environment on transportome genes and on genes documented as indicators of cellular oxidative stress at an organ level. Therefore, we extended our preceding studies employing the same comparative gene expression analysis to focus on genes associated with metabolic gatekeeping, and oxidative stress in spaceflight-exposed pregnant rat mammary glands.

## 2. Materials and Methods

### 2.1. Animals and Treatment Conditions

The research protocol was reviewed and approved by the NASA Animal Care and Use Committee prior to experimentation. The housing conditions, husbandry, and climate controls for the experimental animals were described in detail previously [51,52]. Briefly, time-bred pregnant Sprague-Dawley rats (*n* = 4) were flown aboard the space shuttle (STS-70) from days 11 to 20 of pregnancy, and samples were collected surgically within an hour of the shuttle landing following induction of anesthesia with halothane. The control group of pregnant (*n* = 4) rats was exposed to matching environmental conditions, such as light and temperature, present on the shuttle as described previously [51,52].

### 2.2. Isolation of Total RNA

The methods for RNA extraction using Trizol^®^ Reagent (Invitrogen, Carlsbad, CA, USA), along with assessing concentration (Nanodrop Technologies, Wilmington, DE, USA) and purity (Bioanalyzer, Agilent Inc., Palo Alto, CA, USA) are detailed in a recent publication [50].

### 2.3. RNA Preparation for Microarrays

The manufacturer’s recommended protocols for RNA amplification and biotinylating were followed (NuGEN, San Carlos, CA, USA). Thereafter, samples were hybridized to the Rat 230 2.0 GeneChip^®^ array (Affymetrix, Santa Clara, CA, USA). The microarray data were deposited in the Gene Expression Omnibus (GEO; www.ncbi.nlm.nih.gov/geo, accession no. GSE12132, accessed on 14 March 2022).

### 2.4. Microarray Gene Expression Analysis

The data normalization and transformation using the multichip analysis approach (RMA) are described earlier [50]. Thereafter, the resultant raw data files were imported into R (Bioconductor) for analysis with LIMMA version 3.50.3 [53]. Multiple testing correction (FDR) can be conservative when small sample sizes are involved, leading to higher false negatives [54,55]. To highlight this, for this data set (with *n* = 4 per treatment group) when corresponding *p*-values were adjusted to control for multiple testing in this study, it yielded only one significant gene (GPT2) at a threshold of 0.05 (*q*-value). Given the nature of these treatments (flying pregnant female rats into a low-gravity environment), increasing sample sizes was not an option. Because of these factors, we used unadjusted *p*-value to identify as many significant features as possible considering that data of this kind is limited. Therefore, the differentially expressed genes were filtered using fold change (≥1.2 or ≤1.2) difference in expression and an unadjusted *p*-value ≤ 0.05 was selected. Biological and pathway enrichment analyses were performed using the Database for Annotation, Visualization, and Integrated Discovery [DAVID] (https://www.david.ncifcrf.gov/, accessed on 14 March 2022) resource. The functional clustering, node interactions, and connectivity of differentially expressed transport genes were identified using Revigo (http://revigo.irb.hr/, accessed on 14 March 2022) and Cytoscape (https://cytoscape.org/, accessed on 14 March 2022).

The differentially expressed transmembrane transporters were cataloged into associated families based on the Human Genome (HUGO) organization, and Bioparadigms (www.bioparadigms.org, accessed on 14 March 2022) classification. The selection of oxidative stress probe was based on Human Oxidative Stress collection (Qiagen; Human Oxidative Stress Plus PCR Array) and additional literature [56,57].

### 2.5. In-Vitro Glucose Metabolic Assays

The technique to compute the rate of C-14 glucose oxidation to CO_2_ and incorporation to lipids in-vitro from isoflurane-anesthetized dams was previously described [52]. Briefly, excised mammary gland tissue was sliced into 0.5 mm sections using a handheld microtome (Thomas Scientific, Swedesboro, NJ, USA) and the rate of oxidation of labeled glucose to CO_2_ and glucose incorporation into lipids was calculated and expressed as nmoles of glucose utilized per 100 mg tissue/3 h incubation period. The mean comparisons were performed by Tukey’s test following ANOVA. *p* < 0.05 were considered significant [52].

### 2.6. Quantitative Polymerase Chain Reaction (q-PCR)

We could not independently verify the changes in transcript abundance identified in this study employing the Rat 230 2.0 GeneChip^®^ array as neither the same source total RNA that was used for the array nor any archived tissue samples from the same animals are still available. Notwithstanding this, our earlier studies [49,50,51] from these experimental animals demonstrated excellent concordance of differentially expressed genes between microarray and qPCR data. Some of the SLC transcripts assayed in these earlier studies included, Solute Carrier Family 2, member 1 (SLC2A1, Assay ID Rn00593670_m1), Solute Carrier Family 2, member 4 (SLC2A4, Assay ID Rn00562597_m1), Solute Carrier Family 5, member 1 (SLC5A1, Assay ID Rn00564718_m1), Solute Carrier Family 25, member 4 (SLC25A4, Assay ID Rn01438951_m1), and Solute Carrier Family 25, member 5 (SLC25A5, Assay ID Rn00577177_m1). The endogenous controls used were β_2_-Microglobulin (*B2M*, Assay ID Rn00560865_m1)] and β-Actin (ACTB, Rn00667869_m1) and the relative amounts of target gene expression for each sample in these studies were calculated using the formula 2^−ΔΔCT^ [58,59].

## 3. Results

### 3.1. Functional Enrichment Analysis of Differentially Expressed Genes

**Figure 1:**
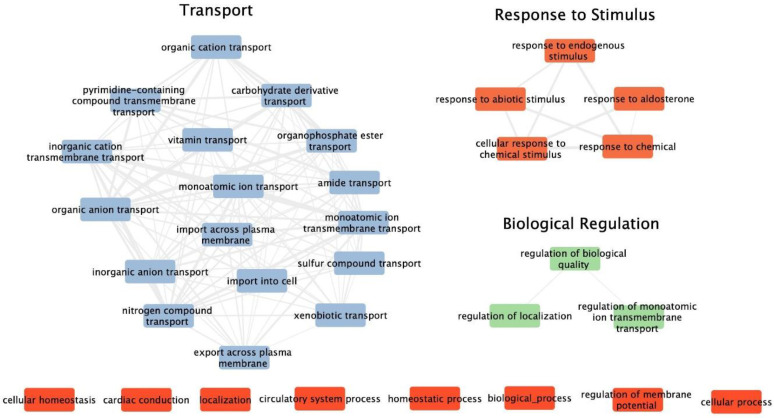
Functional clustering plot showing interactions for the top 100 enriched Gene Ontology (GO) biological processes for differentially expressed genes from our dataset. Individual processes without network connections are indicated at the bottom. Networks were originally constructed in Revigo (http://revigo.irb.hr/, accessed on 14 March 2022) with the small setting selected to reduce the complexity of the network and then modified in Cytoscape. The nodes for the most enriched categories are displayed as livid (transport), red (response to stimuli) and green (biological regulation), while the edges are shown as gray.

The functional categories perturbed by exposure to space are illustrated in Figure 1. The main biological (GO) process enriched included those related to transport, response to stimuli and biological regulation. The connectivity of pathways within the enriched categories is described by functional nodes and edges shared by the statistically significant differentially expressed genes (Figure 1).

### 3.2. Effect of Spaceflight on Solute Carrier (SLC) Membrane Transporter Genes in the Mammary Gland of Pregnant Rats

**Figure 2:**
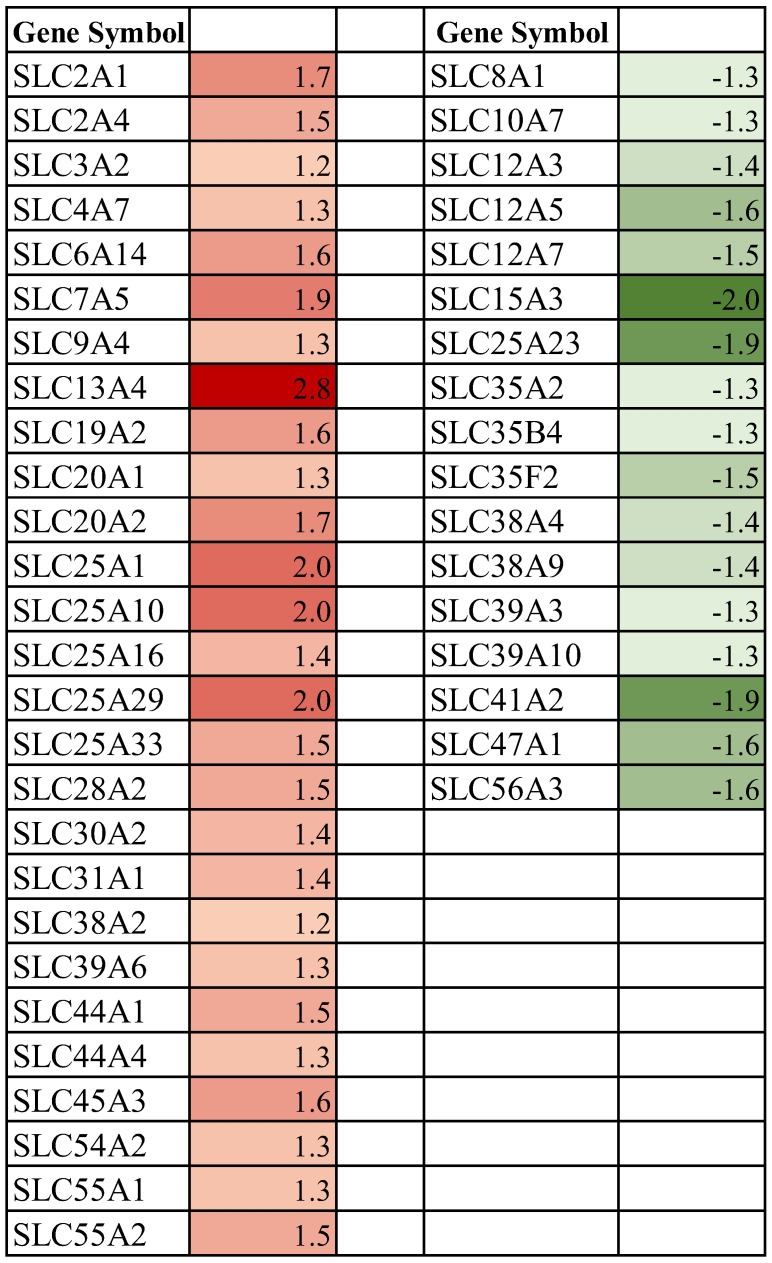
Differentially expressed SLC genes in spaceflight-exposed periparturient rat mammary gland compared to ground-based controls [fold-changes are shown]. Upregulated genes are represented in red (Fold ≥ 1.2, *p* ≤ 0.05), and downregulated are denoted in green (Fold ≤ −1.2, *p* ≤ 0.05).

**Figure 3:**
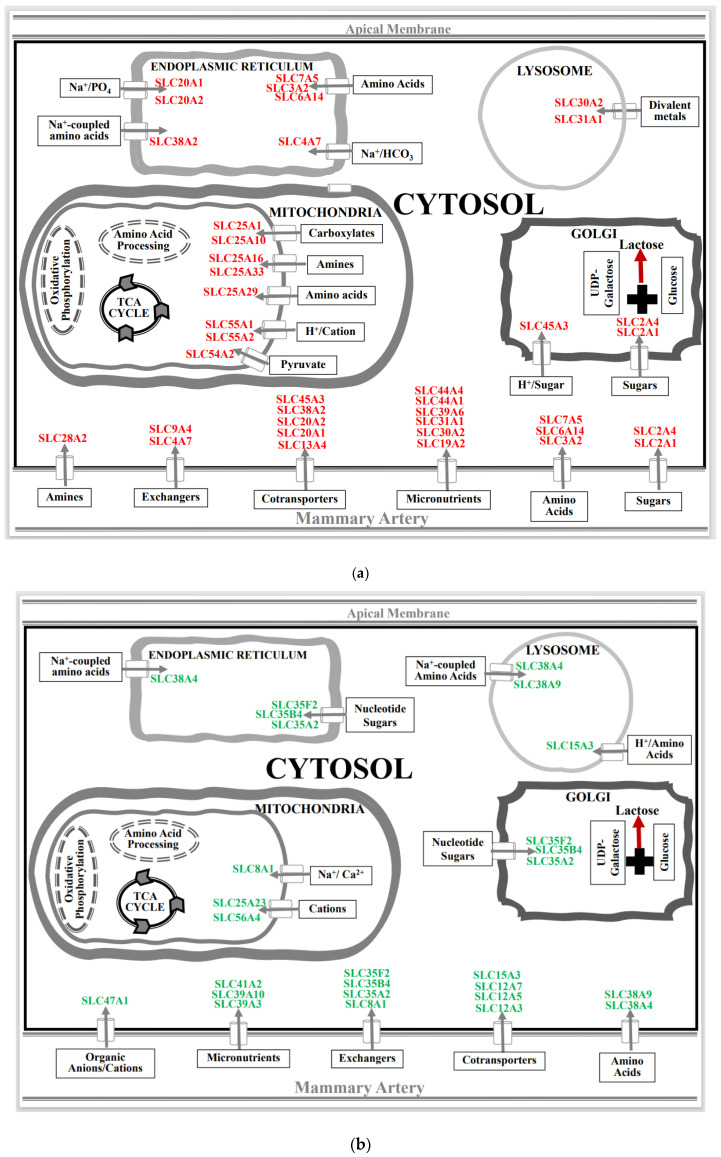
A schematic illustration of metabolite-based clustering of SLC genes at the organelle and cellular levels in spaceflight-exposed periparturient rat mammary gland by expression (*p* < 0.05) pattern, (**a**) induced, and (**b**) suppressed compared to ground-based controls.

The SLC family mediates the passage of a wide array of molecules that play an indispensable role in maintaining cellular homeostasis. These integral membrane proteins are located on the plasmalemma and on the surfaces of the diverse intracellular membranous organelles. Analysis of differentially expressed SLC genes in the mammary glands of pregnant rats exposed to a space environment showed an upregulation (Fold ≥ 1.2, *p* < 0.05) of 27 genes and a downregulation (Fold ≤ −1.2, *p* < 0.05) of 17 genes compared to ground-based controls (Figure 2). Functional clustering using DAVID revealed that the upregulated group was significantly enriched for mitochondrial substrate/solute transporters (Enrichment score 4.4, *p* < 0.01), ion (Enrichment score 3.5, *p* < 0.01), amino acid (Enrichment score 3.16, *p* < 0.01) and sugar transport (Enrichment score 2.13, *p* < 0.01) (Figure 3a). The most highly upregulated gene in this group was SLC13A4 (Fold 2.8, *p* < 0.01) (Figure 2). While the functional clustering of the downregulated SLC genes revealed an enrichment of genes associated with cation, chloride (Enrichment score 5.34, *p* < 0.01), sodium (Enrichment score 3.62, *p* < 0.01), amino acids, and sugar (Enrichment score 2.6, *p* < 0.01) transport (Figure 3b). The highly downregulated gene detected was SLC15A3 (Fold −2.0, *p* < 0.01) (Figure 2).

### 3.3. Effect of Spaceflight on Ionic Channels, ABC, and ATPase Transporter Genes in the Mammary Gland of Pregnant Rats

**Figure 4:**
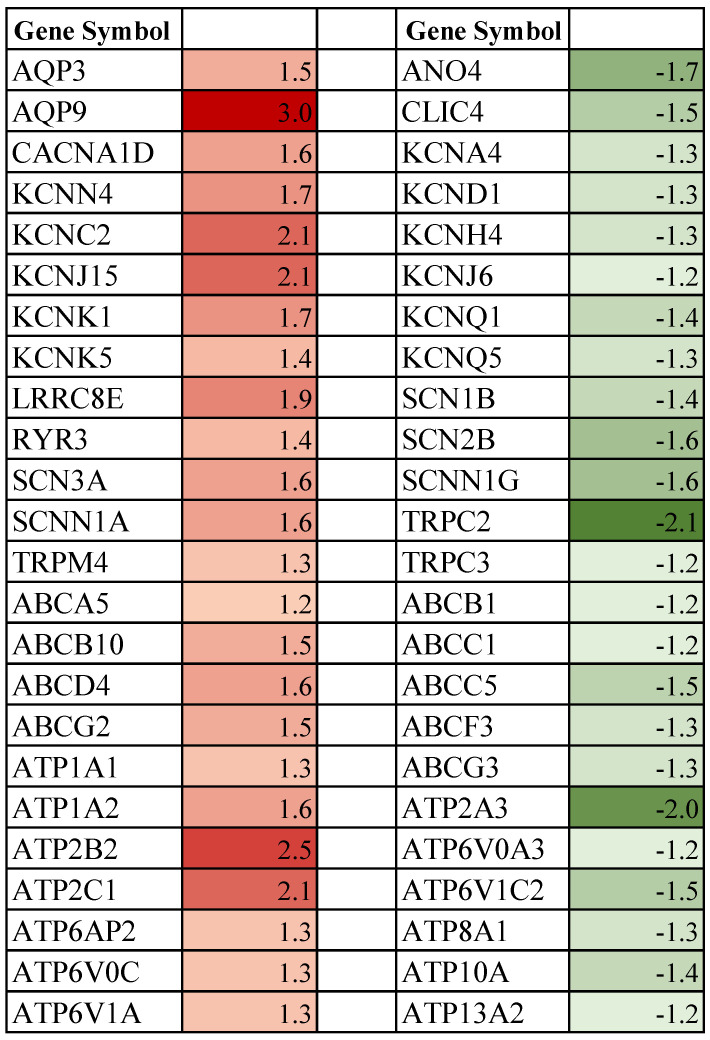
Differentially expressed ion-channel, ABC, and ATPase genes in spaceflight-exposed periparturient rat mammary gland compared to ground-based controls [fold-change values are shown]. Upregulated genes are represented in red (Fold ≥ 1.2, *p* ≤ 0.05), and downregulated are denoted in green (Fold ≤ −1.2, *p* ≤ 0.05).

**Figure 5:**
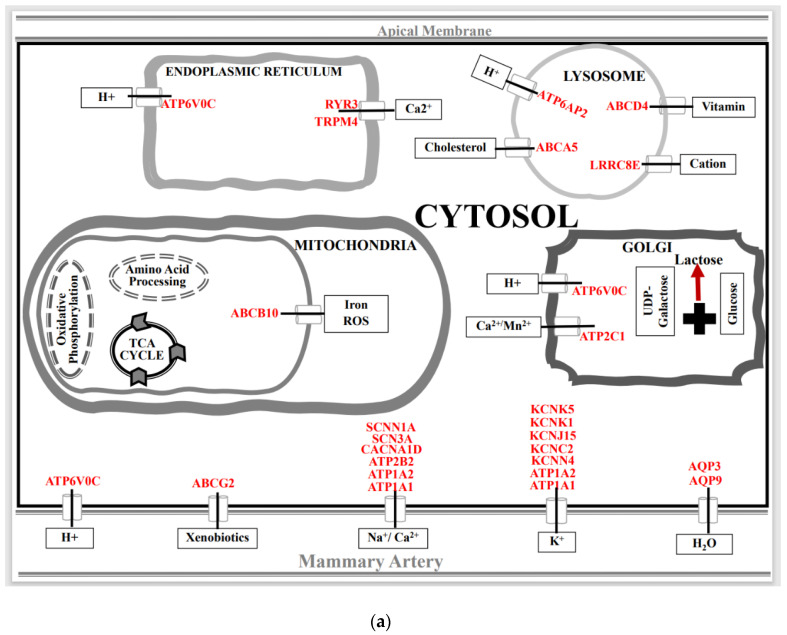

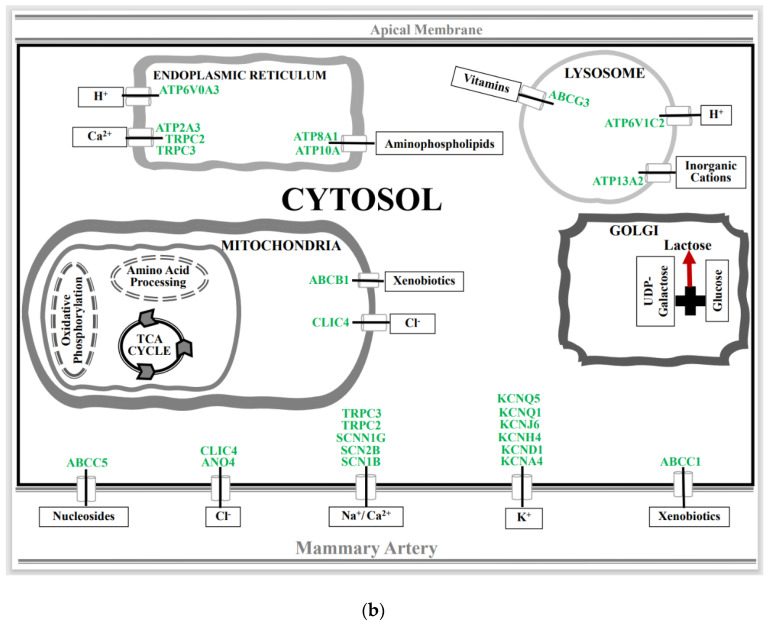
A schematic illustration of metabolite-based clustering of ABC and ATPase genes at the organelle and cellular levels in spaceflight-exposed periparturient rat mammary gland by expression (*p* < 0.05) pattern, (**a**) induced, and (**b**) suppressed compared to ground-based controls.

Ion-selective channels, ABC, and ATPases are essential for maintaining steady-state intracellular ionic balance. We found 13 genes (Fold ≥ 1.2, *p* < 0.05) upregulated and the same number of genes downregulated (Fold ≤ −1.2, *p* < 0.05) from the ion-channel gene family in the pregnant females that were aboard the spaceflight (Figure 4). Functional analysis revealed that genes encoding for water [AQP9 (Fold 3.0)], calcium [CACNA1D (Fold 1.6)], potassium [KCNC2 (Fold 2.1), KCNJ15 (Fold 2.1)], sodium [SCN3A (Fold 1.6), SCNN1A (Fold 1.6)] and chloride [LRRC8E (Fold 1.9)] showed a higher level of expression in the experimental group (*p* < 0.05; Figure 5a). The highly downregulated (*p* < 0.05; Figure 5b) ion transporter genes associated with the transportation of potassium, sodium, and chloride were KCNQ1 (Fold −1.4), SCNN1G (Fold −1.6)] and ANO4 (Fold −1.7), respectively. Comparably, three ABC and six ATPase genes were upregulated (Fold ≥ 1.2, *p* < 0.05), while four ABC and six ATPase genes were downregulated (Fold ≤ −1.2, *p* < 0.05) in the mammary gland post-exposure to space environment (Figure 4 and Figure 5a,b). The most highly upregulated genes in the ABC and ATPase groups were ABCD4 (Fold 1.6 *p* < 0.01) and ATP2B2 (Fold 2.4, *p* < 0.01), respectively. The strongly repressed genes observed in the ABC and ATPase groups were ABCC5 (Fold −1.5, *p* < 0.01) and ATP2A3 (Fold −2.0, *p* < 0.01), respectively.

### 3.4. Effect of Spaceflight on Genes Associated with Cellular Redox Process in the Mammary Gland of Pregnant Rats

**Figure 6:**
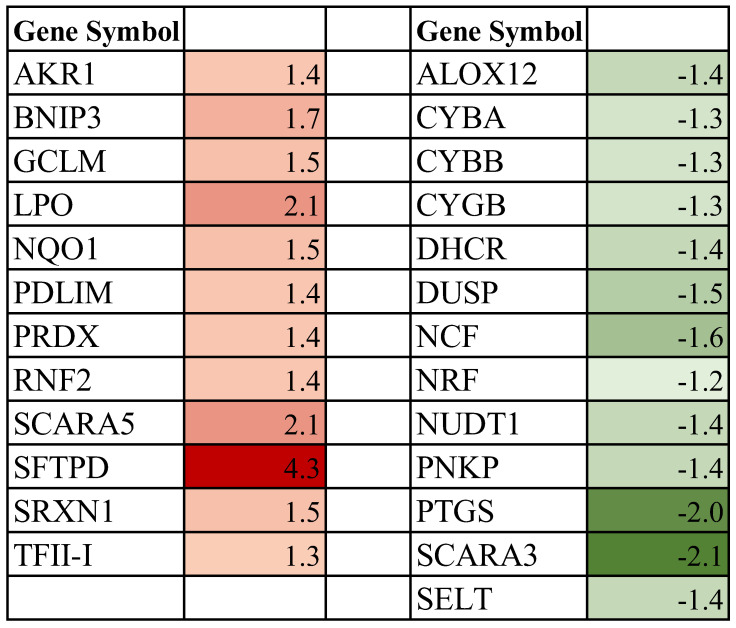
Differentially expressed redox genes in spaceflight-exposed periparturient rat mammary gland compared to ground-based controls [fold-change values are shown]. Upregulated genes are represented in red (Fold ≥ 1.2, *p* ≤ 0.05), and downregulated are denoted in green (Fold ≤ −1.2, *p* ≤ 0.05).

The exquisite balance between the intracellular Reactive Oxygen Species (ROS) and antioxidants is central to maintaining homeostasis. We identified that exposure to space environment induced expression of 12 genes (Fold ≥ 1.2, *p* < 0.05) and downregulated (Fold ≤ −1.2, *p* < 0.05) expression of 13 genes associated with redox balance in the mammary gland of treated pregnant rats (Figure 6). The most highly up- and down-regulated genes in this category were SFTPD (Fold 4.3, *p* < 0.01) and SCARA3 (Fold −2.1, *p* < 0.01), respectively.

### 3.5. Effect of Spaceflight on the Rate of Labeled Glucose Oxidation and Incorporation in Lipids in the Mammary Gland of Pregnant Rats

**Figure 7:**
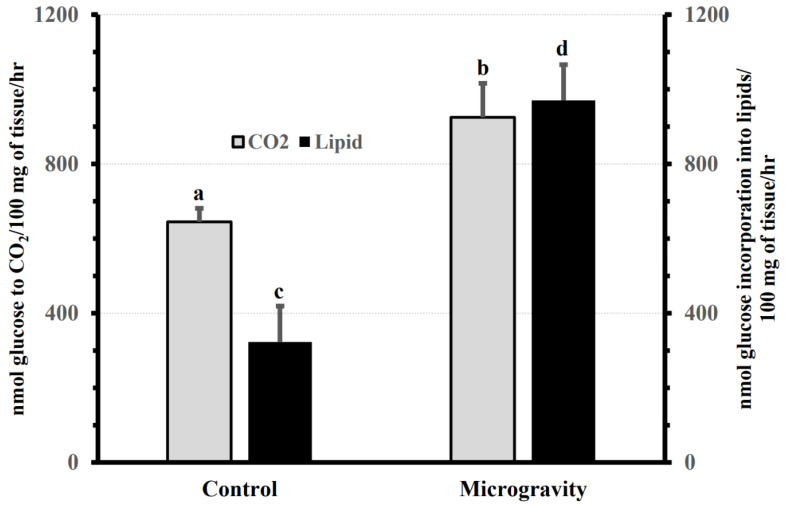
The rate (Mean + SE) of glucose oxidation (Gray bar) and incorporation into lipids (Black bar) in the mammary tissues of microgravity and control rats during pregnancy. Tissue incubation parameters for oxidation and incorporation into lipids were calculated and expressed as nmoles of glucose utilized per 100 mg tissue per 3 h of incubation. Means without a common superscript are significantly different, a, b = *p* < 0.05; c, d = *p* < 0.05. The graph is adapted from previously published data [52].

The rate of glucose oxidation in the mammary gland of spaceflight-exposed rats was significantly (*p* < 0.05) higher per 100 mg of tissue compared to ground-based control animals (Figure 7). Similarly, the rate of glucose incorporation into lipids was approximately three times higher (*p* < 0.05) in the experimental animals than in the mammary glands of pregnant control rats (Figure 7).

## 4. Discussion

Pregnancy modulates the most significant architectural remodeling and functional transition of the mammary gland culminating in the maturation of lobuloalveolar structures that are indispensable for lactation [21,22,23]. In addition, pregnancy not only dictates the profound molecular and tissue evolution of the mammary gland but also is a major determinant of lactational competence [21,22,23]. Neonate’s development, health, and overall growth are primarily dependent on the nutrient contents of the milk, and more importantly, the efficient transfer of the nutrients to the mammary gland [23,60]. Over the last decades, investigators have identified diverse transporters that facilitate the translocation of these nutrients and other molecules across plasma and organelle membranes [26,27,28,31]. These gateways play a critical role in maintaining homeostasis at the cellular and organ levels, including the mammary gland.

### 4.1. Spaceflight and Solute Carrier (SLC) Membrane Transporters

The SLC group is the largest family of transporters with over 400 members identified. The SLC family expedites the passage of a wide array of nutrients and metabolites across the plasmalemma, as well as organelles, and are vital for cellular metabolic homeostasis. Our results show that the expression of multiple putatively functional members of the SLC superfamily is altered in the mammary gland of pregnant rats exposed to the space environment (Figure 2). Our results show that spaceflight-exposure increases the expression levels of the principal transporters of glucose, SLC2A1 and SLC2A4 (*p* < 0.001), in the mammary gland (Figure 2 and Figure 3a). Glucose is not only the major precursor of the dominant carbohydrate of milk, lactose, but also a key determinant of milk yield. As such, sequestration of glucose in the mammary gland commences during pregnancy to meet the neonatal demands of energy. Approximately a 50% increase in the rate of glucose oxidation, as well as ~200% increase in lipogenesis was noted in mammary tissue of rats exposed to microgravity (Figure 7). The current findings of increased expression of both SLC2A1 and SLC2A4 in these experimental animals may provide the biological plausibility to this increased conversion of labeled glucose in mammary tissue [52]. Notwithstanding this, overexpression of SLC2A1 or SLC2A4 or both has been shown to improve glucose utility in GLUT1/GLUT4 transgenic mice [61]. Similarly, we found that the expression of the hexose-proton symporter (SLC45A3) that is capable of transporting both hexose and pentose sugars was increased (*p* < 0.01) in animals exposed to microgravity (Figure 2 and Figure 3a). Vitavska et al. [62] showed that SLC45A3 increased sugar uptake fourfold under a hyperosmolar environment, but the role of SLC45A3 in mammary epithelial cells remains to be elucidated. It is plausible that the upregulation of SLC2A1, SLC2A4, and SLC45A3 in the mammary glands of rats exposed to the space environment facilitates increased glucose utilization, which is consistent with the metabolic findings (Figure 7). However, further studies are needed to confirm this.

Mitochondria are key organelles driving cellular energy homeostasis, and they play a crucial role in ion homeostasis, fatty acid biosynthesis, cellular signaling, apoptosis, and immunity. Accordingly, mitochondrial biogenesis machinery is activated during pregnancy-associated mammogenesis to increase the number of mitochondria per cell necessitated by the demands of lactation [63,64]. Our results show that the organic acid transporters facilitating the transfer of TCA cycle bound citrate (SLC25A1), succinic acid (SLC25A10), and pyruvate (SLC54A2) (Figure 2 and Figure 3) are upregulated (*p* < 0.05) in the mammary gland of experimental rats. Similarly, Ca^2+^/H^+^ antiporters (SLC55A1; SLC55A2), along with the carrier of acylcarnitine’s (SLC25A29) were also induced (*p* < 0.01) in this same group of animals. The other upregulated (*p* < 0.05) mitochondrial genes in experimental animals were ATP/ADP (SLC25A16) translocator and pyrimidine nucleotide (SLC25A33) carrier. From a bioenergetic perspective, our results of increased mRNA expression of carboxylic acid carriers and nucleotide translocases concur with the findings of increased oxidation and fatty acid synthesis reported in the mammary glands of rats exposed to the space environment (Figure 7). Furthermore, other studies have validated that an upregulation of carboxylic acid transporters proportionally increases the uptake of these carboxylates [65,66]. Conversely, the mRNA abundance of iron (SLC56A4), Na^+^/Ca^2+^ (SLC8A1), and ATP-Mg^2+^/Pi exchangers (SLC25A23) were suppressed (*p* < 0.01; Figure 2 and Figure 3) in the spaceflight rats. Magnesium is indispensable for the various enzymatic reactions involved in cellular energetics [67] and dysfunction of SLC25A23 disrupts magnesium-driven ATP production in the mitochondria [67]. Overall, our findings reveal that expression of a myriad of mitochondrial SLC genes is altered in mammary epithelial cells of pregnant rats exposed to space environments.

Amino acids are not only required for protein and nucleic acid syntheses but are a vital source of cellular energy [21,60]. Amino acids are also precursors of various hormones, neurotransmitters, and anaplerotic metabolites [21,60]. Consequently, amino acids play an important role in maintaining cellular and systemic homeostasis. The mammary gland’s demand for amino acids dramatically increases during pregnancy to facilitate the intricate tissue remodeling and to meet the postpartum amino acid, and protein needs of the neonate [23,24]. Therefore, the effectual transfer of amino acids to the mammary gland during pregnancy and lactation is critical since it synthesizes over 90% of polypeptides de novo. Our findings reveal that spaceflight exposure induced (*p* < 0.05) expression of both sodium-dependent (SLC6A14, SLC38A2) and -independent (SLC3A2, SLC7A5) amino acid transporters, while the mRNA abundance of sodium-coupled transporters of neutral (SLC38A4, SLC38A9) amino acids were downregulated (*p* < 0.05) in the pregnant rat mammary gland (Figure 2 and Figure 3). Researchers have shown that SLC7A5 and SLC6A14 are upregulated in several cancers of epithelial origin, including breast cancer [68,69]. The luminal SLC38A4 transfers neutral and cationic amino acids, such as glutamine and alanine, and its atypical expression is also associated with an increased risk of metabolic diseases such as diabetes, in addition to cancers [70]. However, the homeostatic consequence of altered expression of these amino acid transporters in the pregnant mammary gland is unknown and remains to be elucidated.

### 4.2. Spaceflight and Membrane Cotransporters and Exchangers

Cotransporters and exchangers have evolved evolutionarily to support mammary gland development by regulating fluid flow, cell volume and modulating intracellular pH [71]. Our studies show that exposure to spaceflight increases (*p* < 0.01) the expression of sodium-coupled sulfate (SLC13A4), and phosphate (SLC20A1, SLC20A2) symporters in the mammary glands of pregnant rats (Figure 2 and Figure 3a). Sulfate is essential for branching and lobuloalveolar maturation of the mammary gland, as well as fetal morphogenesis [72]. Similarly, phosphate is indispensable for various cellular undertakings from energy storage to the synthesis of biomolecules, and the intracellular uptake of monovalent phosphate (H_2_PO_4_^−^) anion is mediated by SLC20A1 and SLC20A2 [73]. Recently, SLC20A1 was identified as a useful prognostic biomarker for hormone-positive breast cancer, and a higher expression of this membrane transporter is associated with poor prognosis [74]. On the other hand, multiple cation-coupled chloride (SLC12A3, SLC12A5, SLC12A7) symporters were downregulated (*p* < 0.01) in the experimental group (Figure 2 and Figure 3b). The SLC 12 family contributes to ion fluxing and plays a pivotal role in cytosolic acid-base homeostasis [26]. Notably, multiple endoplasmic reticula and Golgi nucleotide sugar antiporters (SLC35A2, SLC35B4, SLC35F2) were downregulated (*p* < 0.05) in the treatment group (Figure 2 and Figure 3b). Broadly, the loss of SLC35 function leads to defects in the glycosylation of proteins, lipids, and aminoglycans [75]. However, more research is needed to better understand the effect of spaceflight on glycoprotein synthesis in the subcellular organelles of the mammary gland.

### 4.3. Spaceflight and Micronutrient Membrane Transporters

Micronutrients play a critical role in cellular, molecular, and metabolic processes required to maintain homeostasis [26,27]. They regulate pivotal biochemical reactions and are indispensable catalysts for innumerable enzymes [26,27]. Among the trace elements, zinc is essential for cellular catalytic, organizational, and regulatory functions [76,77]. Zinc cellular levels are tightly regulated through the synchronized actions of two distinct SLC families; SLC30A and SLC39A that are responsible for the plasmalemmal efflux and influx of Zn^2+^, respectively [76,77]. Our findings reveal that exposure to spaceflight induces the expression of Zn^2+^ importers, SLC30A2 (*p* < 0.001) and SLC39A6 (*p* < 0.05), while concurrently downregulating (*p* < 0.05) SLC39A3 and SLC39A10 that are involved in the cellular acquisition of Zn^2+^ in the pregnant rat mammary gland (Figure 2 and Figure 3). This suggests that the space environment directly impacts zinc homeostasis by dysregulating both influxion and intracellular pooling of Zn^2+^ in the mammary epithelial cells. We also found that SLC31A1 which mediates Cu^2+^ import into the cells was upregulated (*p* < 0.05), whereas choline (SLC44A1, SLC44A4) influxors were suppressed (*p* < 0.05) in rats exposed to space environment (Figure 2 and Figure 3). In mammary epithelial cells, the imported Cu^2+^ is shuttled to the Golgi complex and integrated with a glycoprotein (ceruloplasmin) which is the key trafficker of Cu^2+^ in milk [78]. Growing neonates require vast amounts of choline and the principal source of this choline is milk [79]. Overall, these microelement transporters are not only vital for the highly regulated uptake of trace elements across plasmalemma and intracellular organelles but play a major role in maintaining homeostasis.

### 4.4. Spaceflight and Ion-Channels

Ion channels mediate the influx and efflux of specific inorganic ions and play critical roles in diverse cellular processes from generating membrane potential to acid-base balance to volume regulation [80]. Among the channels, the highly selective aquaporins (AQP) regulate H_2_O fluxes and are vital to osmoregulation and water homeostasis. Among the thirteen named members of the mammalian AQP family, AQP3 (*p* < 0.05) and AQP9 (*p* < 0.001) were upregulated in the mammary glands of spaceflight-exposed rats (Figure 4 and Figure 5a). These two AQPs are identified as aquaglyceroporins that mediate H_2_O and glycerol trafficking [81]. Also, a recent study reveals that AQP9 facilitates monocarboxylates passage into the cytosol and mitochondria [82]. Dysregulation of AQP3 and AQP9 impairs osmoregulation, energy production, and removal of ROS [83,84], which would likely alter mammary function. Among the inorganic ions, we found that the mRNA expression of multiple channels traffickers of the key cations namely, calcium (CACNA1D, RYR3, TRPM4, TRPC2, TRPC3) sodium (SCN3A, SCNN1A, SCN1B, SCN2B, SCNN1G), potassium (KCNC2, KCNN4, KCNJ15, KCNK1, KCNK5, KCNA4, KCND1, KCNH4, KCNJ6, KCNQ1, KCNQ5), and anion, chloride (ANO4, CLIC4, LRRC8E) was altered (*p* < 0.05) in the experimental animals (Figure 4 and Figure 5). The diverse cellular functions of these ligand- and voltage-gated channels are well documented [85,86]. Na^2+^ and K^+^ ions are essential for mammary epithelial cells to facilitate differentiation, proliferation, and organ remodeling during pregnancy [87,88]. Additionally, Na^2+^ and K^+^ channels are key mediators of Na^2+^ and K^+^ efflux into milk during the lactation period [89]. Impaired expression of these Na^2+^-ligand (SCNN1A, SCNN1G), Na^2+^-voltage-gated (SCN3A, SCN1B, SCN2B), K^+^-ligand (KCNN4, KCNJ15, KCNK1, KCNJ6, KCNQ1) and K^+^-voltage-gated (KCNC2, KCNA4, KCNH4, KCNQ5) and the chloride (ANO4) channels triggers pH imbalance, alters osmoregulation, affects cell volume, and enhances metastatic progression of gynecological cancers [90,91]. While, significant advances have been made in understanding the pathophysiology caused by impaired ion shuttling in neurological and cardiovascular systems [80,92], detailed ion channelopathies in the mammary gland remain to be characterized.

### 4.5. Spaceflight and ABC and ATPase Transporters

The ATP-driven ABC-ATPase transport system mediates the shuttling of a plethora of substrates across the internal and external membranes against concentration gradients [93,94]. The ABC-ATPase families are also implicated in translocating phospholipids to maintain the organizational homeostasis of the plasma membrane [93]. Among the ABC family, we found that exposure to spaceflight increased the mRNA expression of ABCB10 (*p* < 0.05) and ABCD4 (*p* < 0.01) in the pregnant rat mammary gland (Figure 4 and Figure 5a). ABCB10 is localized to the inner mitochondrial membrane and is essential for protection against oxidative stress and iron homeostasis [95,96]. On the other hand, ABCD4 effluxes lysosomal-stored cobalamin that is indispensable for both cytosolic enzymes synthesizing polypeptides, as well as TCA cycle-involved mitochondrial enzymes [97].

Among the ATPase transporters, the plasma membrane located ATP1A1 (*p* < 0.05) and ATP1A2 (*p* < 0.001) channels that play a pivotal role in cellular osmotic homeostasis by maintaining the Na^+^/ K^+^ balance was upregulated in rats exposed to the space environment (Figure 4 and Figure 5). Similarly, the plasma membrane cation transporting ATPase, ATP2B2 (*p* < 0.01), Golgi-localized ATP2C1 (*p* < 0.01), and endoplasmic reticulum located ATP2A3 (*p* < 0.05) that play a critical role in calcium homeostasis displayed lower abundance in experimental rats (Figure 4 and Figure 5). Likewise, the expression of multiple subcellular organelle-sited proton ATPases (ATP6AP2, ATP6V0C, ATP6V0A3, ATP6V1C2) that modulate H^+^ fluxes and are critical for maintaining cellular pH homeostasis was altered (*p* < 0.05) in spaceflight-exposed rats. Two members of the phospholipid flippase complex, ATP8A1 (*p* < 0.05) and ATP10A (*p* < 0.01), that translocate glycerophospholipids required for cell membrane stability and permeability were downregulated in experimental animals [98]. Overall, ATPases are not only involved in mammary gland differentiation, but are also indispensable for metabolic function [98,99].

### 4.6. Spaceflight and Cellular Redox Process

Reactive Oxygen Species (ROS) are important for a variety of physiological processes and a tight balance between intracellular prooxidants and antioxidants must be maintained to uphold the physiological state of equilibrium [100,101]. Consequently, The energy-transducing mitochondria are the major source of ROS in a cell, while the cytosol and single-membrane-encompassed organelles do make secondary contributions to the cellular ROS pool [101]. Our results show that some of the major players within the NADPH complex linked to superoxide production (CYBA, CYBB, NCF) and the redox-sensitive transcription factor NRF were downregulated (*p* < 0.05) in the mammary gland of experimental animals (Figure 6). In addition, the key superoxide dismutases, SOD1, SOD2, and SOD3 were also repressed in spaceflight-exposed rats but fell below the statistical cutoff (*p* > 0.05). Low levels of SOD have been previously reported in astronauts returning to Earth, indicating a reduction in oxidation inhibition [102]. On the other hand, several other oxidative stress genes associated with peroxiredoxins (LPO, SFTPD; *p* < 0.01), metal-chelating (RNF2; *p* < 0.01), mitochondria (BNIP3, TFII-I; *p* < 0.01) and cytosol (PDLIM; *p* < 0.05) were induced in the experimental animals (Figure 6). Similarly, several cytosolic (AKR1, NQO1, SCARA5) and mitochondrial (PRDX, GCLM, SRXN1) antioxidative genes were upregulated (*p* < 0.05) in pregnant rat mammary gland with exposure to the space environment. Whereas the expression of other antioxidant cytoprotective genes associated with the nucleus (DHCR7, PNKP), mitochondria (ALOX12, DUSP, PNKP, SELT), and cytosol (SCARA3, NUDT1) were repressed (*p* < 0.05) in experimental rats. SFTPD and SCARA3 are suggested as biomarkers of oxidative stress [103,104] and their dysregulation is associated with metabolic dysfunction [105,106]. Our findings of altered expression of ROS genes concur with earlier studies examining the impact of spaceflight on oxidative stress [107,108]. Importantly, altered redox homeostasis adversely impacts an array of membrane transport pathways. For example, the deleterious effects of ROS on diverse ion channels are well documented [109,110]. These detrimental effects also extend to ATP-driven pumps, as well as ion exchangers, including proton exchangers that are requisite for pH homeostasis [111,112]. Elevated ROS levels directly impact the trafficking of phospholipids leading to alterations in the membrane structure, organization, and stability [113]. Moreover, impaired ROS balance impedes water uptake through the water-selective channels AQP, and as well, damages biomolecules that compromise cellular signaling and structural integrity of mammary tissue [114,115,116]. Therefore, it is important that mitigation strategies are implemented to ameliorate the harmful effects of oxidative stress on both non-ATP and ATP-dependent transporters to protect astronauts’ health as we explore more distant celestial bodies.

To summarize, our results indicate that exposure to the space environment impacts the transportome genes that govern the influx/efflux of ions, nutrients, waste products, and endomembrane-synthesized biomolecules, along with genes related to oxidative balance. It is plausible that the divergence in the abundance of these transporters and oxidative stress-related genes is eliciting many of the systemic and metabolic adaptations observed in the space environment. More notably, the planned manned missions to Mars, the moon or other celestial bodies may place astronauts at greater health risk due to the alterations of these transporter-mediated pathways. Furthermore, the severity of the long-term consequences of deep space exploration on an individual that returns to an environment where gravity has a profound effect on homeostasis remains unknown. Therefore, further studies in animal models are warranted to confirm the present findings, as well as aid in developing countermeasures for the health and productivity of astronauts sent on deep space exploratory missions.

## Data Availability

The data is accessible through the NCBI GEO database, accession no. GSE12132.
